# Thermal Degradation of Glass Fibre-Reinforced Polyamide 6,6 Composites: Investigation by Accelerated Thermal Ageing

**DOI:** 10.3390/polym17040509

**Published:** 2025-02-16

**Authors:** Alessandro Salvi, Francesco Marzullo, Marlena Ostrowska, Giovanni Dotelli

**Affiliations:** 1 Politecnico di Milano, Dipartimento di Chimica, Materiali e Ingegneria Chimica “Giulio Natta”, 20133 Milano, Italy; alessandro.salvi@polimi.it (A.S.); fmarzullo20@gmail.com (F.M.); 2 ABB, 24123 Bergamo, Italy; marlena.ostrowska@it.abb.com

**Keywords:** accelerated thermal ageing, polyamide composites, glass fibre, activation energy, flexural strength, Arrhenius model

## Abstract

Polyamide-based glass fibre-reinforced composites are extensively used in electrical and automotive applications due to their excellent mechanical, thermal, and electrical properties. However, prolonged exposure to high temperatures can lead to significant degradation, affecting their long-term performance and reliability. This study investigates the thermal ageing behaviour of polyamide 6,6 composites containing halogenated flame retardants used for electrical applications. The objective of this research is to evaluate the extent of degradation through accelerated ageing tests and to develop an Arrhenius-type ageing model to predict the long-term performance of these materials. This study examines the effects of thermal ageing at temperatures between 160 and 210 °C on flexural properties and explores the underlying degradation mechanisms. Results indicate that short-term exposure to high temperatures can enhance flexural strength due to annealing effects, which are eventually outweighed by thermal oxidation and increased crystallinity, leading to an increase in brittleness. The derived Arrhenius model, with an activation energy of 93 kJ/mol, predicts a service life of approximately 25 years at 80 °C, but a significantly shorter one at 130 °C. These findings underscore the importance of considering thermal ageing effects in the design and application of PA66 composites in high-temperature environments.

## 1. Introduction

Polyamide-based glass fibre-reinforced (PA-GF) composites are widely utilised in both electrical and automotive applications thanks to their exceptional mechanical, thermal, and electrical properties. In the electrical sector, these materials are ideal for components like accessory boxes, levers, and terminal covers in low-voltage circuit breakers, where they are exploited for their stiffness, impact resistance, and thermal stability [1]. In the automotive sector, PA66 and PA6 composites are commonly employed in interior components, engine parts, and bodywork due to their high stiffness, excellent impact and wear resistance, and strong chemical resilience [2,3]. However, prolonged exposure to high temperatures typical of these applications inevitably leads to degradation, which can significantly affect the long-term performance and reliability of these materials.

In fact, polymeric materials commonly undergo changes over time, a process often referred to as ageing. The degradation of polymers typically involves a partial breakdown of the material into fragments that remain relatively large but are smaller than the initial polymer chains [4,5,6]. This fragmentation is a key aspect of the ageing process, which can be triggered by prolonged exposure to various environmental factors such as heat, light, moisture, oxygen, and chemicals [7,8].

The primary degradation mechanisms in PA66 composites during thermal ageing include the following:

▪ Hydrolysis: a chemical degradation mechanism favoured by high temperatures, which occurs when water molecules penetrate the polyamide matrix and break the polymer chains. This can lead to a decrease in molecular weight, plasticisation of the matrix, and a reduction in mechanical properties, such as stiffness and strength [9,10,11]. The fibre/matrix interface is particularly susceptible to hydrolysis, as water molecules tend to accumulate in this region [12,13,14].
▪ Thermo-oxidation: another chemical degradation mechanism that involves the reaction of oxygen with the polyamide matrix at elevated temperatures [15,16,17,18]. This process can lead to chain scission, the formation of free radicals, and the creation of various degradation products, such as carbonyl, chromophoric groups, or peroxides [19,20].
▪ Fibre/Matrix Debonding: Ageing can lead to interfacial debonding (or mismatch), which weakens the stress transfer between the matrix and the fibres, resulting in a decline in mechanical properties [21]. Factors like poor interfacial adhesion [22], the presence of water [23,24], the presence of voids [25], and the differing coefficients of thermal expansion between the fibres and the matrix [26] can contribute to debonding.
▪ Physical Ageing/Plasticisation: Water absorption can cause plasticisation of the polyamide matrix, leading to a decrease in stiffness and an increase in ductility [27,28]. This occurs because the water molecules disrupt the hydrogen bonding between the polymer chains, increasing their mobility [10,22,29]. While plasticisation can improve toughness in the short term [30], prolonged exposure to moisture can lead to more severe degradation, like hydrolysis [31].

Accelerated ageing tests are valuable tools for investigating the long-term effects of thermal ageing on GFRPs (Glass Fibre-Reinforced Plastics), such as PA66 composites. These tests simulate real-world operating conditions in a shorter timeframe, enabling the assessment of material degradation and the development of lifetime prediction models.

Research has primarily focused on thermal–oxidative ageing, hygrothermal ageing, and the influence of other ageing-related factors, such as manufacturing processes and interfacial engineering.

Shu et al. [17] studied the thermal–oxidative degradation mechanism demonstrating that early-stage thermal–oxidative ageing in PA6 involves molecular crosslinking, temporarily enhancing mechanical strength and viscosity. However, prolonged exposure leads to chain scission and oxidative byproduct formation, reducing mechanical performance and melting temperature, while increasing crystallinity.

Alexis et al. [32] extended their investigation to fatigue durability, demonstrating that thermal ageing at 200 °C for 500 h had a significant impact on fatigue life and cyclic indicators such as hysteresis energy density and cyclic mean strain rate. Moreover, ageing led to stiffening and embrittlement in short glass fibre-reinforced PA6/PA66 composites, with lower fibre content exacerbating the degradation.

Similarly, Yarar et al. and Sahin et al. [33,34] studied the effects of short-term thermal ageing on PA6 composites. Ageing decreased tensile and flexural strength by up to 10%, while flexural modulus was improved. Moreover, they studied the tribological properties of the material, showing the detrimental effect of ageing that significantly increased wear volume and wear rate.

The role of interfacial engineering in mitigating such degradation was also highlighted by Rudzinski et al. [35], who were able to improve the thermal–oxidative stability of PA66 composites through advanced fibre sizing. Their work showed that the inclusion of silane, polyurethane, and epoxy/acrylate film formers enhanced interfacial adhesion, mitigating yellowing and preventing fibre separation during ageing.

From a circular economy perspective, Eriksson et al. [31,36] considered the reprocessing of aged materials by studying the mechanical properties of recycled glass fibre-reinforced PA66 under thermal ageing conditions. The research showed that thermal ageing did not considerably change the tensile strength and modulus, while the elongation at break decreased significantly, particularly with increased recycling of the material.

Hygrothermal ageing, which combines the effects of heat and moisture, has also been a focal point of research. Ksouri et al. [37] investigated PA6 and GF-reinforced PA6 exposed to immersion at 90 °C, observing significant reductions in tensile properties and glass transition temperature. The damage, caused by water absorption and plasticisation, escalated with prolonged exposure, leading to phenomena like crazing and yellowing.

Bergeret et al. [38] similarly reported drastic declines in mechanical performance during hygrothermal ageing, attributed to chain scission and water penetration at the fibre/matrix interface.

Li et al. [39] explored the long-term effects of hydrothermal ageing on GF-reinforced PA6 composites, identifying chain scission and post-crystallization as primary degradation mechanisms that weakened the fibre–matrix interface and reduced tensile strength.

The influence of hydrothermal ageing on wear and tribological behaviour of GF-PA6 composites was also investigated by Khakbaz et al. [40], whose findings revealed that while glass fibres improved wear resistance, ageing weakened tensile strength and increased strain at break due to water absorption.

Further, Wang et al. [41] compared the long-term hygrothermal ageing resistance of glass fibre-reinforced PA and PET composites. They found that GF/PA composites absorbed water at a faster rate and reached higher saturation moisture content compared to GF/PET composites. The ageing also led to a decrease in the tensile strength and heat distortion temperature of both types of composites, but the effect was more pronounced in GF/PA composites, emphasising the material-specific vulnerabilities of polyamides.

Despite the extensive literature on the thermo-oxidative ageing of PA-GF composites, to the best of our knowledge, there is a lack of studies focusing on materials engineered for high-temperature applications, such as those incorporating flame retardants, which are critical for ensuring thermal stability and fire resistance in demanding environments.

Therefore, this study focuses on a specific type of glass fibre-reinforced polyamide 6,6 composite containing halogenated flame retardants, which is commonly used for electrical components working at moderately high temperatures.

This research investigates the thermal ageing behaviour of this material and performs accelerated ageing tests at temperatures between 160 and 210 °C, with detailed characterisation of its mechanical, thermal, and chemical properties to evaluate the extent of degradation. An Arrhenius-type ageing model is developed to determine the activation energy of the degradation process and establish a temperature–time relationship, offering a predictive framework for assessing the long-term performance and reliability of the composite in high-temperature applications. Activation energy estimations are ultimately compared with results obtained from modulated thermogravimetry and correlated to the hypothesised degradation mechanism.

## 2. Materials and Methods

This study focused on a glass fibre-reinforced polyamide composite commonly used for low-voltage circuit breaker components working at moderate to high temperatures. The composite, from now on referred to as “PA-GF”, is based on a polyamide 6,6 matrix, containing 25 wt% short glass fibre reinforcement, and utilising a brominated flame retardant. The material was supplied as both granules and injection-moulded specimens produced in accordance with ISO 178:2019 [42], measuring 80 × 10 × 4 mm. The thermal behavior of the composite was analyzed via Thermogravimetric Analysis (TGA) (Figure S1), while its elemental composition was determined using Inductively Coupled Plasma Optical Emission Spectroscopy (ICP-OES) and X-ray Fluorescence (XRF) (Table 1 and Figure S2, respectively). After moulding, samples were conditioned under controlled humidity and temperature to ensure uniform moisture absorption. For specific tests, samples were dried at 90 °C for 3 h and then stored in a desiccator for 24 h to evaluate the impact of moisture on their mechanical properties.

Thermal ageing was conducted in a drying oven (Binder WTC, BINDER GmbH, Tuttlingen, Germany) to simulate long-term exposure to elevated temperatures. Four different temperatures were selected: 160, 180, 200, and 210 °C, all below the glass transition temperature (Tg) of the material (260 °C). Specimens were aged for periods up to 1080 h, with exposure times at each temperature determined based on an assumed Q_10_ factor of 2, implying that the degradation rate doubles for every 10 °C. The specific ageing times for each temperature are reported in Table 1.

Flexural properties were measured using a three-point bending test setup as specified in ISO 178:2019 [42]. Testing was performed on an Instron 5569 universal testing machine equipped with a three-point flexural apparatus and a span length of 64 mm. A loading speed of 5 mm/min was applied after an initial preload of 2 N was reached. Flexural strength, modulus, and strain at break were calculated as an average from stress–strain curves obtained after testing at least five specimens per sample. Baseline tests were conducted on unaged and moisture-conditioned samples to establish reference values for comparison.

To model the effects of thermal ageing, an Arrhenius-based approach was used to describe the relationship between ageing time, temperature, and degradation of flexural properties. The model is governed by Equation (1):

(1) $$k\left(T\right)=A\cdot e^{\frac{-Ea}{RT}}$$

where

▪ *k*(*T*) is the temperature-dependent rate of property degradation [hour^−1^].
▪ *A* is the pre-exponential factor [hour^−1^].
▪ *Ea* is the activation energy [J/mol].
▪ *R* is the universal gas constant, equal to 8.314 [J/mol/K].
▪ *T* is the absolute temperature [K].

The activation energy for degradation was calculated by plotting the natural logarithm of the degradation time required to reach a specific property threshold against the inverse of the absolute temperature. A threshold corresponding to the 80% of the initial flexural strength has been taken as the endpoint, in agreement with the requirements set by ABB for the specific application of the composite in low-voltage circuit breakers.

Thermogravimetric analysis (TGA) was performed using a Waters—TA Instruments TGA 550 to investigate the thermal degradation behaviour of the samples and to determine the activation energy through modulated thermogravimetry (MTG). The experiments were conducted between 30 and 600 °C, with a modulated heating rate of 5 °C/min, under air atmosphere.

Thermal properties were analysed by differential scanning calorimetry (DSC) using a Mettler Toledo DSC821e device (Mettler-Toledo GmbH, Greifensee, Switzerland) Heating tests were conducted under air with a ramp rate of 20 °C/min. The degree of crystallinity was calculated from the measured melting enthalpy values taking the inorganic content into account.

Molecular changes were monitored by Fourier Transform Infrared Spectroscopy (FT-IR) in attenuated total reflection mode (ATR) with a Perkin-Elmer Frontier spectrometer in the range of 4000 to 380 cm^−1^. Spectra were recorded with 16 scans at a resolution of 1 cm^−1^. The ATR-FTIR spectra obtained were normalised for comparison and the characteristic absorption peak were examined to identify chemical changes caused by thermal ageing.

**Figure 1:**
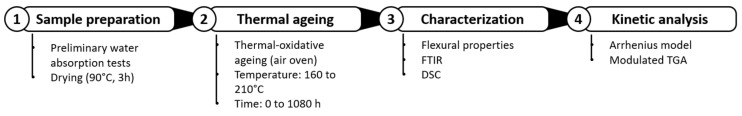
Overview of the experimental procedure: (1) sample preparation; (2) thermal–oxidative ageing; (3) characterisation; (4) kinetic analysis.

An overview of the experimental procedure is provided in Figure 1. Images of the test specimens can be found in the Supplementary Materials (Figures S5 and S6), along with pictures of the testing equipment (Figure S7).

## 3. Results and Discussion

### 3.1. Preliminary Tests: Water Absorption

Preliminary tests were conducted to evaluate the sensitivity of the composite to water absorption and its impact on mechanical properties. Specimens of PA-GF were exposed to a controlled environment at 25 °C and 60% relative humidity (RH) for periods of 0, 8, and 24 days. Flexural tests were performed after each exposure period to assess changes in mechanical behaviour.

**Figure 2:**
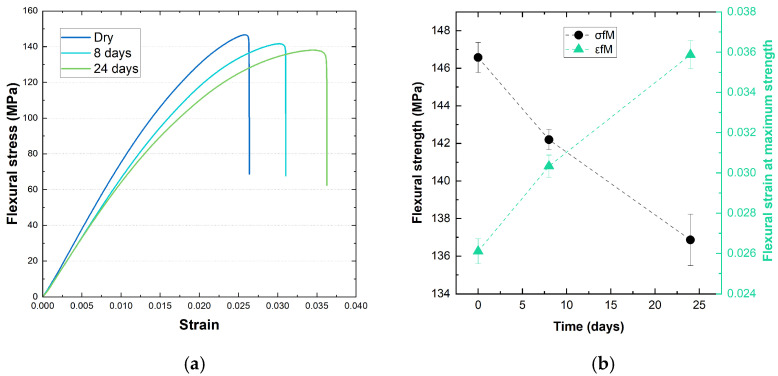
Flexural stress–strain curves (**a**) and trends of flexural properties (**b**) for PA-GF samples obtained by three-point bending test of specimen 0, 8, and 24 days after drying, upon exposure to humidity at 25 °C and 60% RH.

The results reported in Figure 2 show that water absorption led to a decrease in flexural modulus and strength for both materials. After 8 days of exposure, PA-GF exhibited a reduction in flexural modulus of approximately 8%, which increased to 12% after 24 days. Despite these declines in stiffness, the material displayed an increase in strain at maximum flexural strength, indicating a plasticising effect caused by moisture uptake.

This enhancement is attributed to the inherent water sensitivity of the polyamide 6,6 matrix, where polar amide groups can coordinate with water molecules, particularly in the amorphous regions, characterised by a higher diffusivity [43,44,45]. This interaction plasticises the polyamide 6,6 by disrupting hydrogen bonding, increasing flexibility but decreasing strength and stiffness, as highlighted by the 7% decline in flexural strength after 24 days of exposure to moisture.

### 3.2. Flexural Tests

Given the significant moisture sensitivity of the composites, as evidenced by preliminary testing, all mechanical tests on aged samples were conducted on specimens that had been previously conditioned in a desiccator for 24 h. This step guaranteed that the observed effects of thermal ageing were not affected by any residual moisture in the samples.

**Figure 3:**
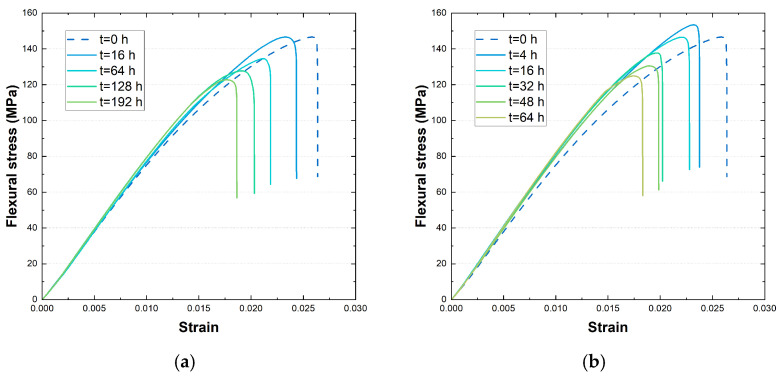
Flexural stress–strain curves for PA-GF samples aged at 180 °C (**a**) and 200 °C (**b**) at different ageing times, compared to the unaged specimen.

Flexural stress–strain curves obtained for the samples aged at 180 °C and 200 °C are reported in Figure 3 for comparison. Similar trends are obtained for the other samples, which are reported in the Supplementary Materials (Figure S3). From the summary of the main flexural properties reported in Table 2, it is evident that ageing significantly impacts the flexural strength (σ_fM_) and flexural strain at maximum strength (ε_fM_) of the material, albeit with distinct kinetic patterns for each property.

Specifically, specimens consistently exhibit a decrease in ε_fM_ and σ_fM_ across all ageing temperatures compared to dry, unaged specimens.

An exception to this trend is the initial increase in flexural strength for short ageing times at higher temperatures (e.g., 4 h at 200 °C, in Figure 3b). This positive effect suggests annealing of the polyamide 6,6 matrix, likely due to residual stress relaxation and potential changes in the crystalline structure [43,46,47]. Stress annealing can be attributed to thermal stresses induced during injection moulding, where the material’s skin solidifies faster than the core, inducing tensile stresses. High-temperature ageing can relax these stresses, increasing σ_fM_. The change in crystalline structure is further explored in the differential scanning calorimetry analysis presented in Section 3.5.

On the other hand, both ageing duration and temperature appear to have little influence on the slope of the curves, leading only to a slight increase in the flexural elastic modulus E_f_ from approximately 7200 MPa (unaged samples) to a maximum of 8000 MPa for samples aged for longer times at higher temperatures.

The deterioration in mechanical properties can be ascribed to a combination of factors within the polyamide 6,6 matrix and at the fibre–matrix interface.

Firstly, the dehydration of water molecules coordinated with polar amide groups in both crystalline and amorphous phases, particularly relevant in the early stages of ageing [44,45,47]. Secondly, the progressive degradation of polyamide 6,6 macromolecules due to thermal ageing, especially at higher temperatures and durations, weakens the entanglement of polymer chains and reduces mechanical properties. Additionally, interfacial phenomena, such as thermal expansion mismatch, can contribute to the loss of flexural properties, particularly at high temperatures. This mismatch, coupled with polymer degradation, weakens the load transfer between fibres and the matrix, leading to the formation of micro-voids and cracks at the interface [37,46,48].

Moreover, it is worth noting that the empirical Q_10_ = 2 kinetic factor previously considered for the thermal ageing duration appears to be valid only for shorter durations at higher temperatures.

### 3.3. Arrhenius Model

Flexural strength data gathered from mechanical testing at different ageing times and temperatures (Table 2) have been used to model a lifetime prediction method through the Arrhenius Equation (1), which can be linearised into Equation (2):

(2) $$\ln\left(k\right)=\ln\left(A\right)-\frac{Ea}{RT}$$

The threshold endpoint selected for the determination of the Arrhenius plot has been set at 80% of flexural strength retained from the material with respect to the initial condition (unaged samples). This value has been chosen considering the acceptability range of mechanical properties indicated by ABB for the use of these materials in low-voltage circuit breakers.

We introduce the following state function:

(3) $$\sigma_{\mathrm{fM}}^{80\%}=k_{i}\left(T_{i}\right)\cdot t_{i}$$

where

- *k_i_* represents the rate of property loss at each ageing condition *i*.
- *t_i_* [h] and *T_i_* [K] denote the ageing time and temperature at which a 20% reduction in flexural strength occurs.

The Arrhenius equation and the state function can be combined to derive Equation (4), where B is a constant factor.

(4) $$\ln\left(t_{i}\right)=B+\frac{Ea}{RT_{i}}\;$$

**Figure 4:**
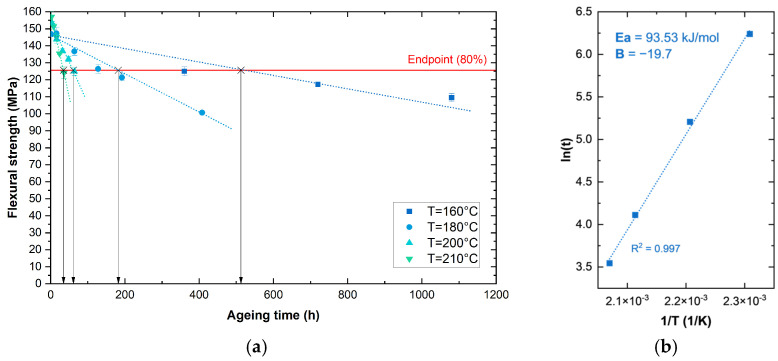
Flexural strength trends for PA-GF samples aged at different times and temperatures (**a**), and Arrhenius plot obtained by setting the endpoint value at 80% of the unaged σ_fM_ (**b**).

Such an equation can be plotted by extrapolating the four values of *t_i_* and *T_i_* obtained from the mechanical tests. Specifically, the value of t_i_ has been determined by linear interpolation of the experimental points, as reported in Figure 4a. The obtained Arrhenius plot is depicted in Figure 4b.

From the Arrhenius law, an activation energy of 93.5 kJ/mol was determined, which is consistent, albeit slightly higher, with values reported in the literature. For instance, research by Jung et al. [49] identified activation energy values in the range of 80–90 kJ/mol by applying an Arrhenius model to the tensile strength retention of aged PA66 composites.

Considering that the material under analysis is currently being used in circuit breakers where it can be exposed to temperature peaks of up to 130 °C, an approximated, conservative estimation of its failure time (i.e., the time necessary to reach a 20% decline in flexural strength) can be calculated using Equation (4). The results are summarised in Table 3.

The results highlight how PA-GF is a suitable material for components exposed to a maximum continuous temperature of 80 °C, which can guarantee its performance for up to 22 years. However, prolonged exposure to higher temperatures can significantly affect the service life of the components.

Moreover, it is worth noting that the activation energy determined by isoconversional methods (such as Ozawa–Flynn–Wall or Kissinger–Akahira–Sunose), ranging from 150 to 190 kJ/mol depending on the specific composite [49,50], is reportedly higher than that obtained from the Arrhenius-based methods. To further verify this trend, the modulated thermogravimetric method has been applied to both materials and reported in the next section.

### 3.4. Modulated Thermogravimetry

In this section, we present the extrapolation of kinetic parameters obtained through modulated thermogravimetry (MTG) for the material under analysis. MTG is a technique that applies an oscillatory temperature program to obtain continuous kinetic information during decomposition and volatilization reactions [51]. By using a sinusoidal temperature modulation, MTG allows for the determination of activation energy and other kinetic parameters throughout the entire decomposition process.

**Figure 5:**
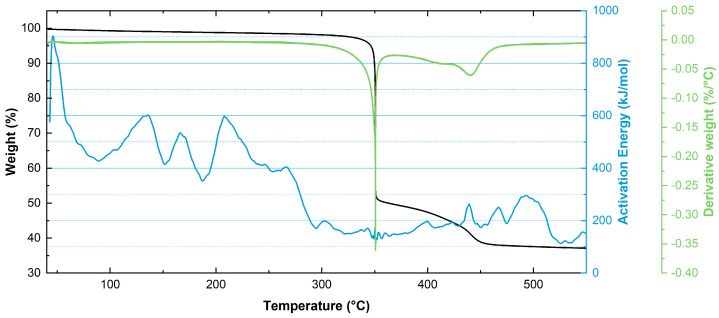
Modulated thermogravimetry analysis, displaying the TGA curve in black, the DTG curve in green, and the calculated activation energy in blue.

The MTG results are depicted in Figure 5. The activation energy (Ea) at low weight loss (5–10%) highlights the initial breakdown of the polymer network. In this phase, the activation energy ranges from 90 to 175 kJ/mol, with an average value of approximately 151 kJ/mol. At these early stages, the thermal degradation of polyamides is supposedly driven by the scission of weaker bonds within the polymer structure, such as the N-alkylamide bond (CH2-NHCO) and the following decomposition of the formed amide group into NH_3_ and CO_2_ [52,53,54,55]. As the temperature increases and the degradation progresses, a gradual rise in Ea is observed, reaching its maximum around 300 kJ/mol, likely due to the further scission of the polyamide backbone and the presence of the flame retardant [56].

The activation energy values obtained with MTG are in good agreement with those reported in the literature using isoconversional methods [49,56,57,58]; however, there is a substantial difference with the Ea values obtained from the Arrhenius model. This difference, already highlighted by the previous literature [49], could be attributed to the different degradation mechanisms involved at the lower temperatures of accelerated ageing tests and could lead to an overestimation of service life by TGA-based methods.

### 3.5. Differential Scanning Calorimetry

DSC analysis of dried PA-HF samples revealed a primary melting peak starting at approximately 246 °C and peaking at 258 °C, consistent with the material’s Tg of 260 °C and with its processing temperature of 275° C reported in the technical sheet. The enthalpy of fusion (ΔH*_m_*) was calculated as 22.06 J/g. Using Equation (5), which accounts for the standard crystalline enthalpy for PA66 (ΔH^0^*_m_* = 196 J/g) and for the solid residue fraction (*f* = 32.96% from TGA), the crystallinity ($\chi_{c}$) of the unaged material was determined to be 16.79%.

(5) $$\chi_{c}=\frac{{\Delta H}_{m}}{{\Delta H}_{m}^{0}\left(1-f\right)}\times 100\;$$

**Figure 6:**
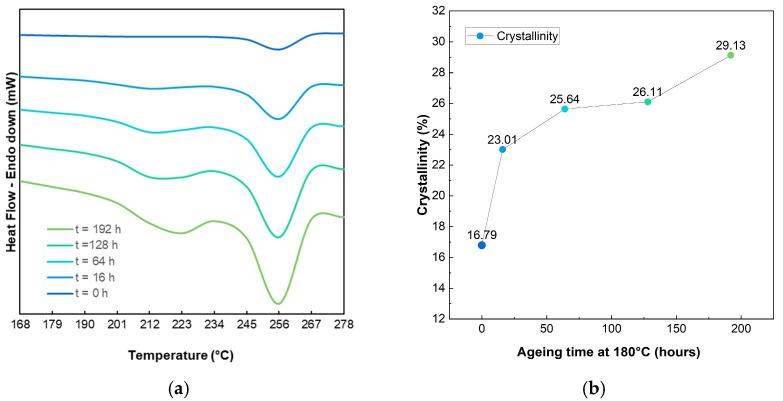
Qualitative DSC curves for PA-GF samples aged at 180 °C for different ageing times, compared to the unaged specimen (**a**); changes in crystallinity for samples aged at 180 °C (**b**).

For aged specimens reported in Figure 6, a secondary melting peak emerged, increasing in intensity and shifting with ageing time and temperature. For example, the secondary peak temperature increased from 208 °C to 221 °C for samples aged at 180 °C for 16 to 192 h, respectively. Similar trends were observed at other ageing temperatures when the ageing time increased, as shown in the DSC plots reported in SM (Figure S8). The primary melting peak, however, remained stable, ranging between 256 °C and 259 °C.

This behaviour aligns with literature findings on polyamide 6,6 [59,60,61] and polyamide 6,6 composites [35,62,63], where thermal ageing induces microstructural changes. Oxidation and thermal exposure cause chain scission in the amorphous phase, allowing shorter chains to recrystallise into new lamellae. Initially, these newly formed lamellae have lower melting points, but prolonged ageing promotes lamellar growth, raising their melting temperature and enthalpy. Crystallinity calculations confirmed this trend, increasing from 16.79% (unaged) to 23.01% after 16 h at 180 °C and stabilising around 25.88% for intermediate ageing times. At 192 h, crystallinity reached 29.13%, indicating higher chain mobility and lamellar growth during prolonged exposure.

### 3.6. Fourier Transform Infrared Spectroscopy

**Figure 7:**
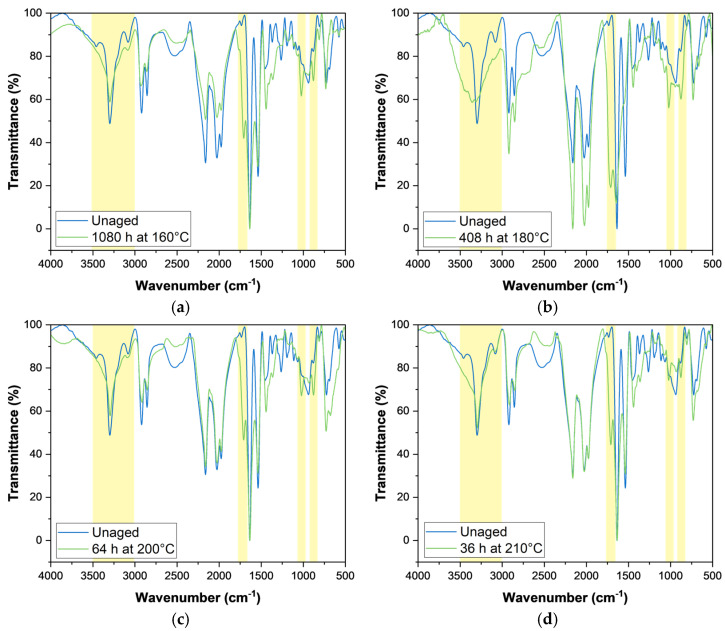
FTIR spectra of unaged and thermally aged PA66 at various conditions: (**a**) 1080 h at 160 °C, (**b**) 408 h at 180 °C, (**c**) 64 h at 200 °C, and (**d**) 36 h at 210 °C. The highlighted regions of the spectra are those more affected by thermal ageing.

The ATR-FTIR spectra in Figure 7 compare the unaged sample with those aged for the longest durations at each temperature, highlighting several key chemical and structural changes.

The increase in absorbance around 1710–1760 cm^−1^, observed across all aged samples, indicates the formation of carbonyl-containing species, such as ketones, aldehydes, or carboxylic acids, as a result of oxidative degradation. Additional evidence is found in the broadening and reduced intensity in the 3000–3500 cm^−1^ region.

In the unaged sample, this region displays two distinct peaks: a sharp band around 3300 cm^−1^, attributed to the free N-H stretching vibrations of the amide groups, and another band near 3080–3200 cm^−1^, corresponding to hydrogen-bonded N-H stretching within the polyamide structure. These peaks are characteristic of the polyamide backbone and reflect its strong hydrogen bonding network.

Upon ageing, significant changes occur in this region, including the disappearance of the two distinct peaks and the broadening of the entire band. This behaviour suggests disruption of the hydrogen bonds within the polymer structure, consistent with the early stages of thermal degradation, during which weaker bonds, such as N-alkylamide bonds (CH_2_-NHCO), undergo scission. This effect is particularly evident in the sample aged at 180 °C for 408 h (Figure 7b), corresponding to the harshest ageing condition among those investigated, as reflected in its pronounced impact on mechanical properties.

Notably, the increased absorbance at ~1020 cm^−1^ suggests the presence of Si-O-Si stretching vibrations, likely resulting from the increased exposure of glass fibres at the polymer surface as the matrix degrades. Similarly, the absorption band in the 800–840 cm^−1^ range, attributed to aromatic C-H bending, becomes more pronounced in aged specimens. This is likely due to the migration of flame-retardant additives, such as polybrominated diphenyl ethers (PBDEs), to the surface during ageing. Supporting evidence includes the concurrent increase in absorbance around 1050 cm^−1^ (C-O stretching of esters) and 750 cm^−1^ (C-Br bonds), both characteristic of PBDE.

Lastly, the broad band around 2500 cm^−1^, typically associated with moisture, is less prominent in the aged specimens. This suggests that surface modifications caused by ageing have reduced the composite’s ability to absorb water.

These spectral changes are consistent across all temperatures studied, confirming that thermal ageing follows a common degradation pathway. Higher temperatures primarily accelerate these processes without altering the underlying mechanisms.

## 4. Conclusions

The results of this study demonstrate how the ageing process affects the structural and mechanical properties of glass fibre-reinforced polyamide 6,6 with brominated flame retardants.

Ageing significantly reduces the flexural strength and strain at maximum flexural strength of the material, with longer durations and higher temperatures exacerbating this decline. Initial improvements in flexural strength at short ageing times, attributed to stress relaxation and annealing, are eventually outweighed by the effects of thermal oxidation, chain scission, and increased crystallinity.

DSC analysis revealed an increase in crystallinity with ageing, driven by chain scission in the amorphous phase and the formation of new crystalline phases. This growth in crystalline regions contributes to the observed brittleness and reduced ductility in the aged material, directly impacting its flexural properties.

FTIR analysis confirmed chemical degradation during ageing, with the appearance of carbonyl peaks indicating oxidative processes and chain scission further contributing to the embrittlement and decline in mechanical performance observed in aged specimens.

The Arrhenius-based lifetime prediction model determined an activation energy of 104.2 kJ/mol, estimating service lifetimes of approximately 25 years at 90 °C but significantly shorter at 130 °C. Modulated thermogravimetry revealed higher activation energy values (151 kJ/mol), reflecting differences in degradation mechanisms during accelerated ageing and thermal decomposition.

Overall, while the composite exhibits favourable long-term stability under moderate temperatures, its use in high-temperature applications must be carefully evaluated, as frequent exposure to heat spikes may severely reduce its durability.

## Figures and Tables

**Table 1:** Ageing times applied for each temperature. The duration of each ageing was determined a priori assuming a Q_10_ factor of 2.

	Ageing Time [Hours]				
	t1	t2	t3	t4	t5
160 °C	360	720	1080	-	-
180 °C	16	64	128	192	408
200 °C	4	16	32	48	64
210 °C	2	8	16	24	36

**Table 2:** Flexural strength (σ_fM_) and flexural strain at maximum strength (ε_fM_) of PA-GF samples at different ageing times and temperatures. The results are reported as an average of at least five measurements, and the standard deviation is negligible.

	Ageing Time [h]	T = 160 °C	T = 180 °C	T = 200 °C	T = 210 °C
	t0	146.9	146.9	146.9	146.9
σ_fM_	t1	125.0	147.2	153.3	157.0
	t2	117.3	136.7	143.9	151.4
	t3	109.5	126.4	137.0	145.0
	t4	-	121.3	132.1	135.4
	t5	-	100.6	124.9	124.1
	t0	0.026	0.026	0.026	0.026
ε_fM_	t1	0.024	0.023	0.024	0.024
	t2	0.022	0.021	0.022	0.023
	t3	0.021	0.019	0.020	0.020
	t4	-	0.017	0.018	0.019
	t5	-	0.016	0.019	0.018

**Table 3:** Activation energy (Ea), logarithm of the pre-exponential factor (B), and failure times evaluated at 130 °C and 90 °C according to the Arrhenius model.

Ea	B	Failure Time at 130 °C	Failure Time at 80 °C
93.5 kJ/mol	−19.7	3706 h (≈155 days)	193,031 h (≈22 years)

## Data Availability

The raw data supporting the conclusions of this article will be made available by the authors on request.

## Supplementary Materials

The following supporting information can be downloaded at: https://www.mdpi.com/article/10.3390/polym17040509/s1, Figure S1: Thermo-gravimetric graph from 0–900 °C showing two main degradation peaks, the first due to polyamide 6,6 matrix thermal degradation between 350 °C and 450 °C, the second at 572 °C can be associated with ASN degradation or CO and CO_2_ evolution; Figure S2: XRF peaks showing the presence of BFR and Zinc, as well as a weak peak in blue that can be attributed to calcium, quantitative considerations can’t be made due to machine low sensitivity for lower energy emitting elements; Figure S3: Flexural stress-strain curves for PA-GF samples aged at 180, 190, 200, 210 °C (b) at different ageing times, compared to the unaged specimen; Figure S4: Cross section view of the fracture surface for PA-GF where it is possible to notice the brittle fracture of the dried and aged for 192 h at 180 °C (picture on the left). It is possible to notice the darkened layer of degradation products on the surface of the specimen; Figure S5: Type of specimens adopted for the flexural tests, with measures according to the standard ISO 178-1 (80*10*4 mm); Figure S6: Test specimens after thermal ageing at 200 °C and mechanical testing, showing progressive darkening due to oxidation with increasing ageing duration. The darkening is evident despite the natural grey color of the unaged specimen; Figure S7: Pictures of the experimental equipment adopted; Figure S8: DSC curves obtained from the first heating of aged PA-GF samples at a heating rate of 25 °C/min showing the evolution of the secondary melting peak for every aging condition; Table S1: ICP-OES weight percent of the main element in PA66+25% GF fillers and additives.
