# Supplementary material for: Thermal Degradation of Glass Fibre-Reinforced Polyamide 6,6 Composites: Investigation by Accelerated Thermal Ageing
Source: Polymers (Basel). 2025 Feb 16;17(4):509. doi: 10.3390/polym17040509 (PMC11859150; doi:10.3390/polym17040509)
Supplement: Supplementary file 1 [file polymers-17-00509-s001.zip › polymers-3412477-supplementary.pdf]

Supporting Materials

Thermal degradation of glass fiber reinforced polyamide 6,6 composites: investigation by accelerated thermal ageing

Alessandro Salvi [1,*], Francesco Marzullo [1], Marlena Ostrowska [2] and Giovanni Dotelli [1]

[1] Politecnico di Milano, Dipartimento di Chimica, Materiali e Ingegneria Chimica "Giulio Natta", Milano, Italy
[2] ABB, Bergamo, Italy
* Correspondence: alessandro.salvi@polimi.it

Unaged Material Characterization

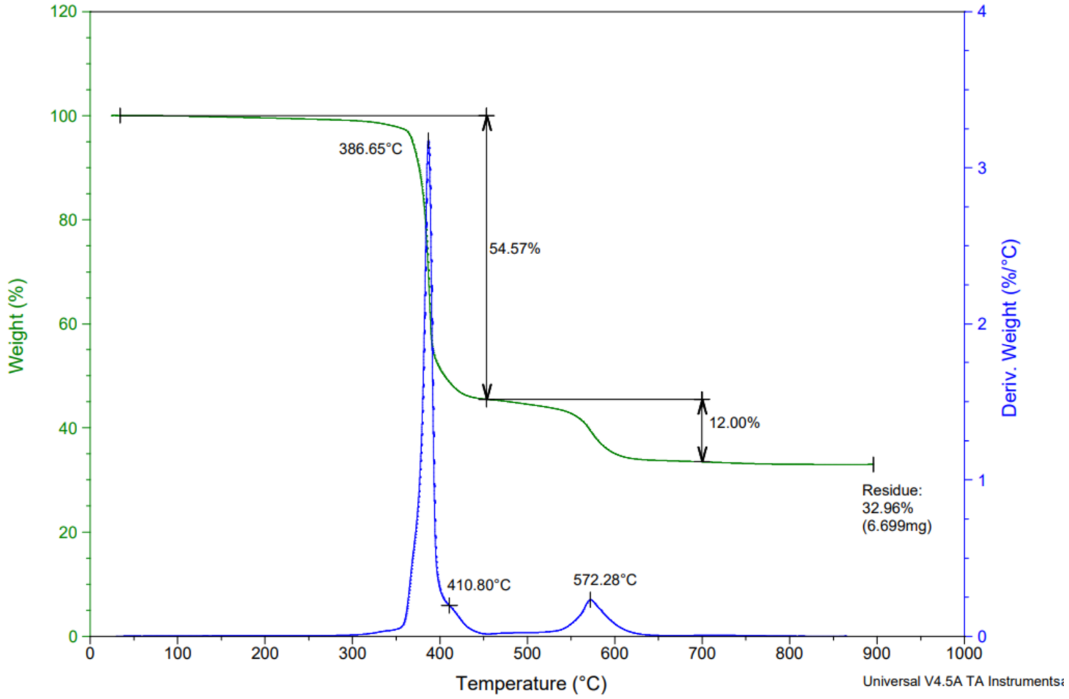

Figure S1 - Thermo-gravimetric graph from 0-900 °C showing two main degradation peaks, the first due to polyamide 6,6 matrix thermal degradation between 350 °C and 450 °C, the second at 572 °C can be associated with ASN degradation or most CO and CO2 evolution.

PA66+25% GF	wt%	Error %
**Al**	1.67	±0.1
**B**	1.07	±0.1
**Ca**	4.39	±0.2
**Mg**	0.42	±0.05
**P**	0.03	±0.01
**Si**	5.24	±0.2
**Ti**	1.46	±0.1

Table S1 - ICP-OES weight percent of the main element in PA66+25% GF fillers and additives

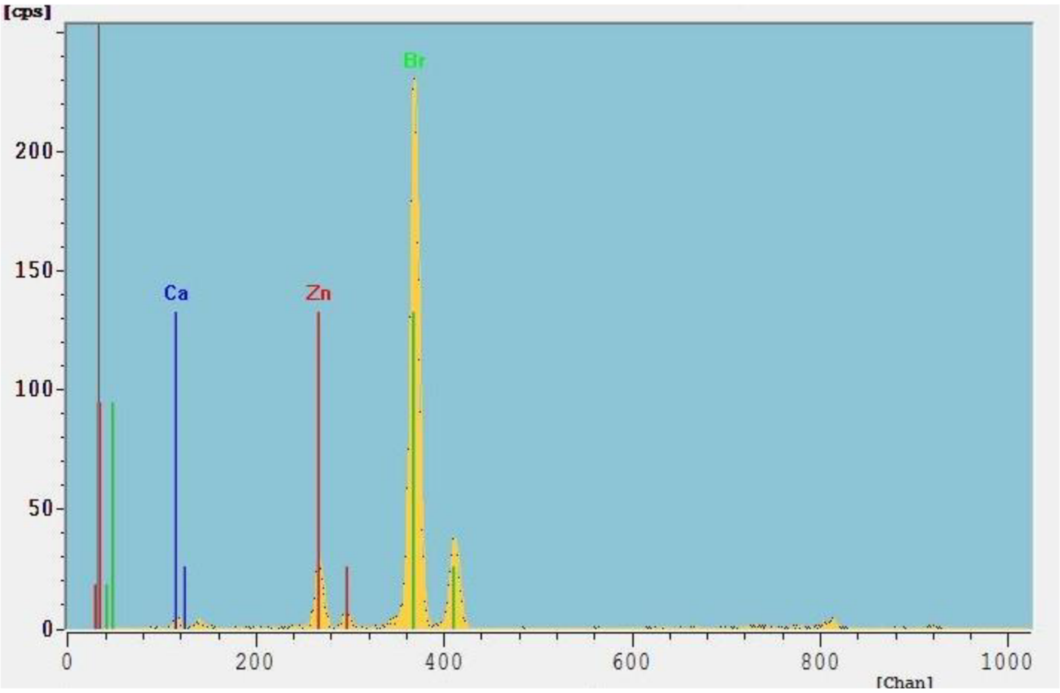

Figure S2 - XRF peaks shows the presence of BFR and Zinc, as well as a weak peak in blue that can be attributed to calcium, quantitative considerations can't be made due to machine low sensitivity for lower energy emitting elements

Flexural tests

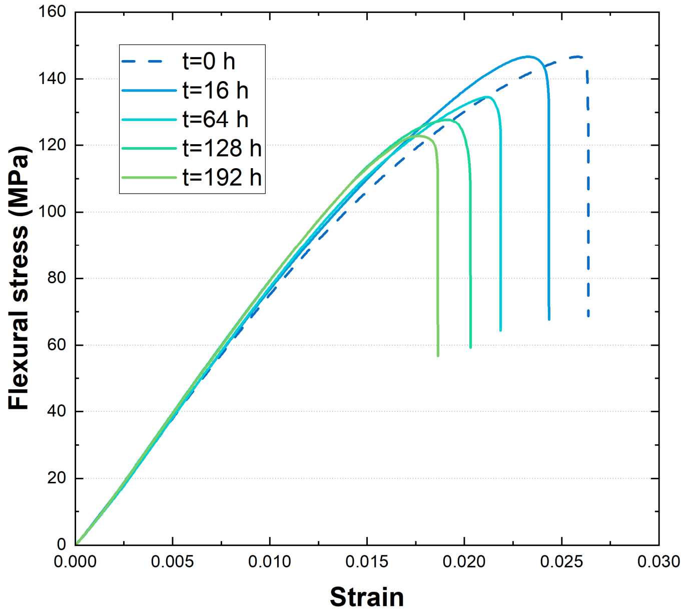

Flexural Strain-stress curve at 180°C

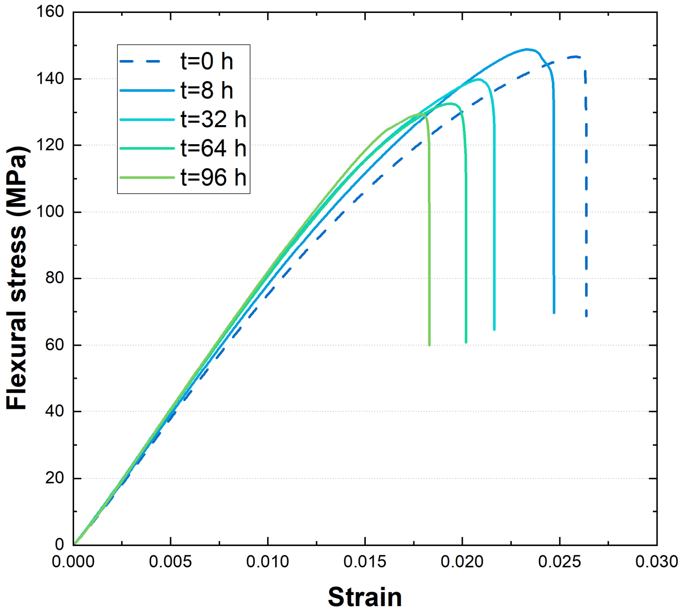

Flexural Strain-stress curve at 190°C

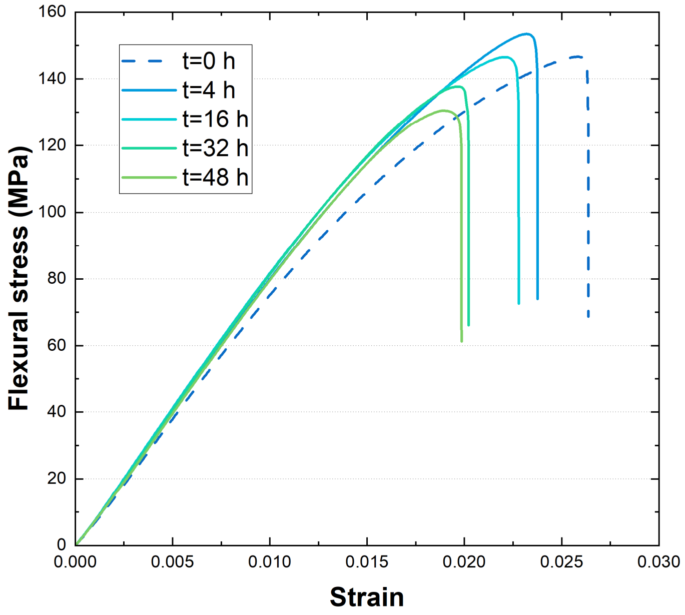

Flexural Strain-stress curve at 200°C

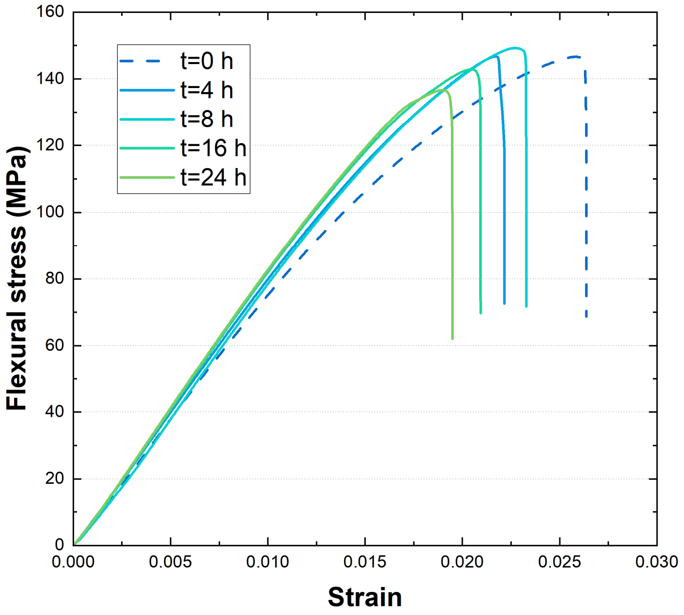

Flexural Strain-stress curve at 210°C

Figure S3 - Flexural stress-strain curves for PA-GF samples aged at 180, 190, 200, 210 °C (b) at different ageing times, compared to the unaged specimen.

Optical Microscope images

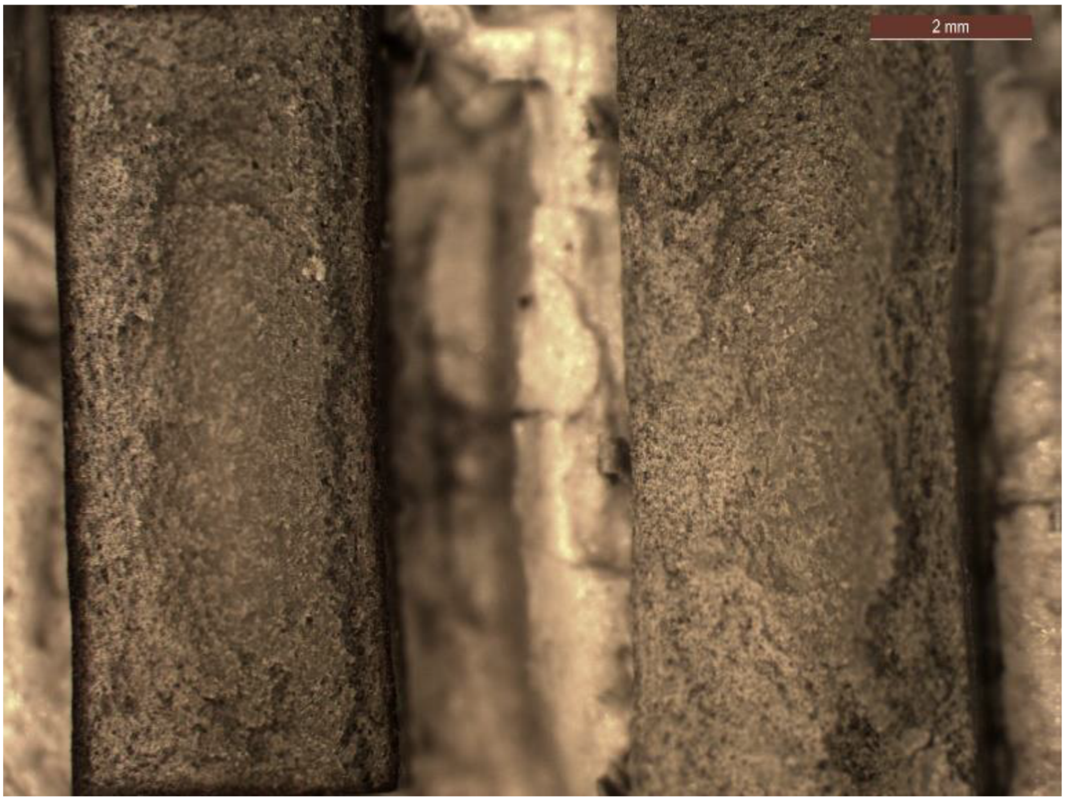

Figure S 4 - Cross section view of the fracture surface for PA- GF where it is possible to notice the brittle fracture of the dried and aged for 192h at 180 °C (picture on the left). It is possible to notice the darkened layer of degradation products on the surface of the specimen.

Images of the specimens

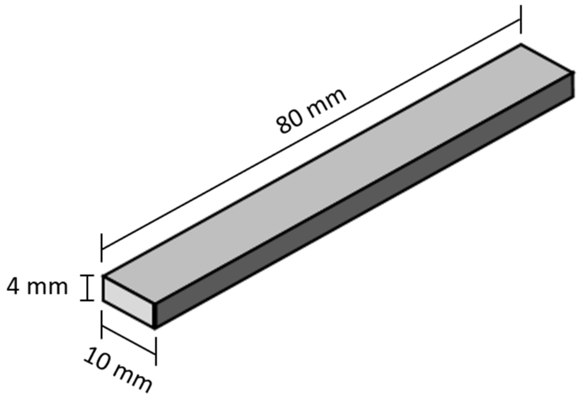

*Figure S 5 – Type of specimens adopted for the flexural tests, with measures according to the standard ISO 178-1 (80*10*4 mm)*

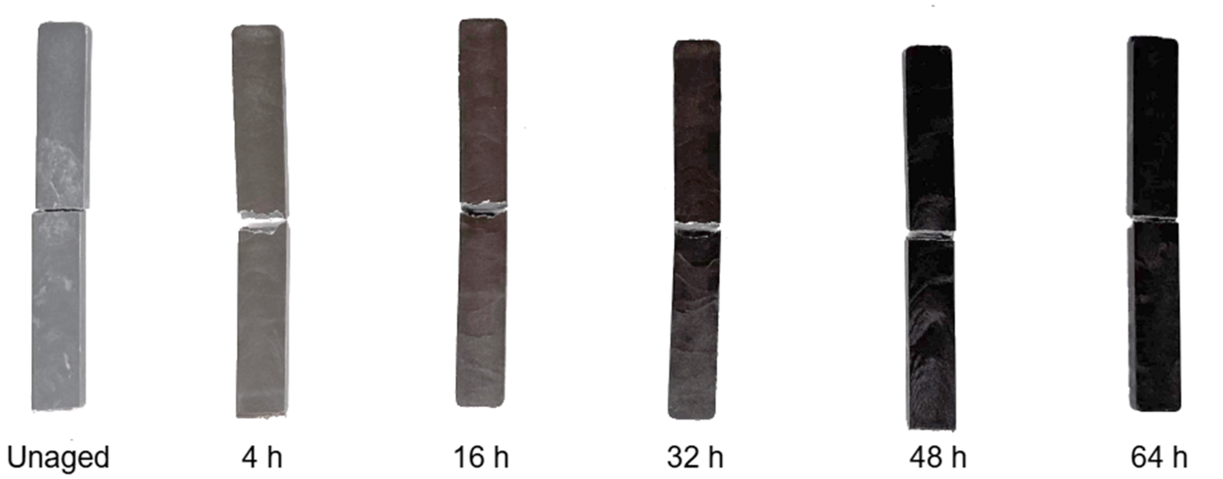

Figure S 6 - Test specimens after thermal ageing at 200°C and mechanical testing, showing progressive darkening due to oxidation with increasing ageing duration. The darkening is evident despite the natural grey color of the unaged specimen

Images of the experimental equipment

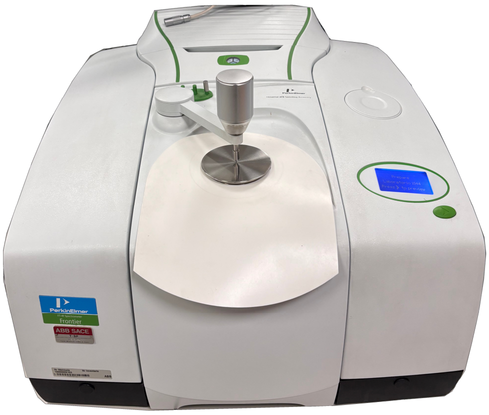

Perkin Elmer Frontier FTIR spectrometer

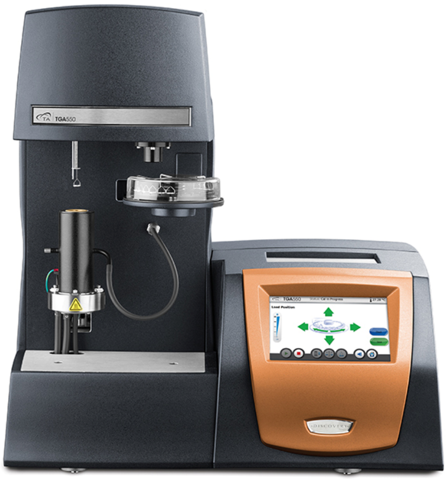

Waters – TA instruments Discovery TGA550

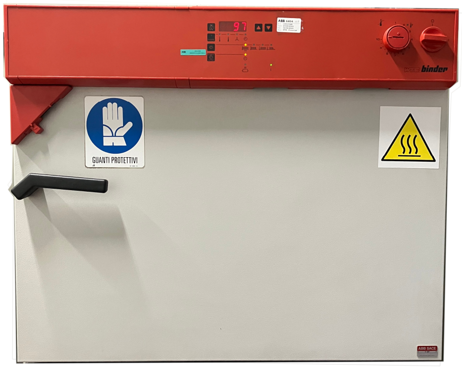

WTC Binder drying oven

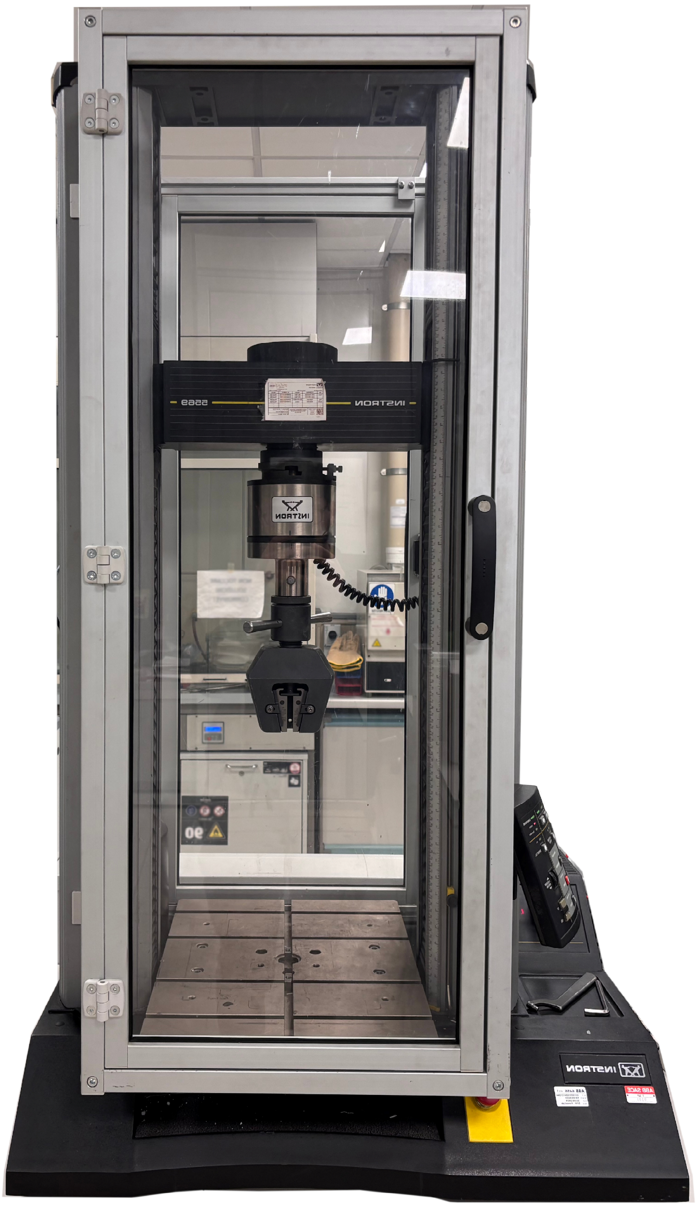

Instron 5569 universal testing machine

Figure S 7 - Pictures of the experimental equpement adopted

Differential Scanning Microscopy

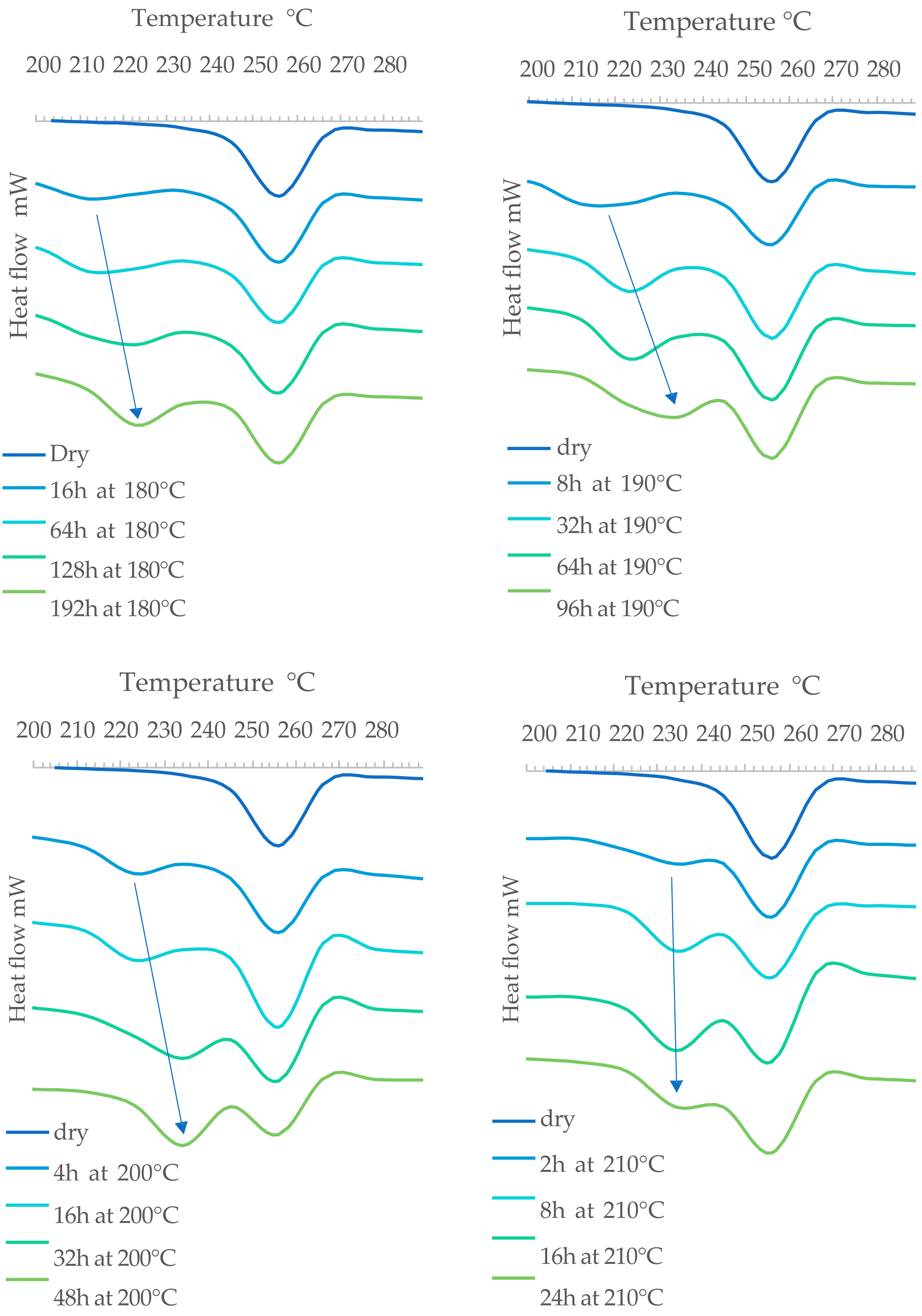

Figure S 8 - DSC curves obtained from the first heating of aged PA-GF samples at a heating rate of 25 °C/min showing the evolution of the secondary melting peak for every aging condition.
